# Gender differences in the measurement of pharmacists’ job satisfaction

**DOI:** 10.1186/s12960-018-0297-5

**Published:** 2018-07-31

**Authors:** Manuel J. Carvajal, Ioana Popovici, Patrick C. Hardigan

**Affiliations:** 1 Department of Sociobehavioral and Administrative Pharmacy, College of Pharmacy, Nova Southeastern University, 3200 South University Drive, Fort Lauderdale, FL 33328-2018 United States of America; 2 College of Medicine, Nova Southeastern University, 3200 South University Drive, Fort Lauderdale, FL 33328-2018 United States of America

**Keywords:** Gender disparities, Job-related preferences, Job satisfaction, Pharmacist workforce

## Abstract

**Background:**

Men and women choose different levels of commitment in their careers and at home. Compared to men, women value the significance of tasks performed and social relations more and earnings less. The objective of this study was to explore whether male and female pharmacists show the same levels of satisfaction overall and with key facets of their job, whether overall satisfaction is associated with satisfaction with 12 key facets of pharmacists’ jobs, and whether this association is similar for men and women.

**Methods:**

The study used self-reported survey data collected from a random sample of licensed pharmacists practicing throughout the United States. The sample consisted of 436 males and 300 females. Pearson correlation coefficients were calculated to assess the association between overall job satisfaction and its key components. The 13 job satisfaction indices and the Pearson correlation coefficient values were compared by gender.

**Results:**

Women were consistently more satisfied than men. Variations in overall job satisfaction were at best accompanied by moderate variations in the 12 job satisfaction facets, raising concerns about the validity of configuring a composite index from multiple indices of satisfaction.

**Conclusion:**

The results of this study can be used by healthcare managers and policymakers to facilitate communication, enhance teamwork, and promote a better allocation of scarce resources. Since men and women responded differently to various facets of their jobs, a constant set of rewards and stimulants may not be equally effective for both genders as employers transform the workplace to more adequately meet practitioners’ needs and increase their productivity.

**Electronic supplementary material:**

The online version of this article (10.1186/s12960-018-0297-5) contains supplementary material, which is available to authorized users.

## Background

The job paths that people take along their career trajectories depend on individual differences, available openings, and existing constraints [1]. When pharmacists assess their current jobs versus alternative employment opportunities, they examine options with multiple characteristics including salary and benefits, location, work conditions, and potential for advancement, among others. These characteristics appear in fixed bundles, and pharmacists seldom are able to choose only the ones they value and/or eschew those they do not like. For example, a job that pays well may demand unattractive work schedules, a promotion may entail moving to an undesirable location, or a position that carries more prestige may be accompanied by numerous stressful episodes. Choices invariably require a metric for weighing and trading off advantages and disadvantages.

The congruency between the kind of work that pharmacists do and the conditions under which they do it, on the one hand, and their idea of a “perfect job,” on the other hand, measures the extent of their job satisfaction [2] and has received worldwide recognition as a proxy for professional utility [3–6]. It is a multidimensional concept that compares the reality of a work environment to workers’ desires and expectations, allowing social and administrative pharmacists to analyze the well-being generated from experiences in a position and/or work setting.

The idea of a “perfect job” is influenced by perceptions, attitudes, and opinions developed from values and social cues that affect pharmacists of both genders differently, or are interpreted differently. Men and women tend to choose dissimilar levels of commitment in the career and home portions of their lives in response to their socially defined, differentiated involvement with childcare and household responsibilities [7, 8]. They also assign unequal weights to identical job characteristics [9, 10]; compared to men, women are likely to value the significance of tasks performed and social relations at work more and consider earnings as a less important aspect of their job. Thus, gender often mediates perceived levels of satisfaction [11].

Job satisfaction indices may be measured in two ways. The first, called a facet-free item, focuses on an aggregate satisfaction scale without reference to specific aspects imbedded into it; the second way, called a facet item, is derived as a composite measure from various indices of satisfaction with key aspects of a job [12]. Using an aggregate measure is easier and avoids methodological problems related to assigning weights to the various facets of one’s job, accounting for different frames of reference, and ensuring that all relevant areas comprising job satisfaction are identified [13], but it has been shown to overestimate satisfaction and underestimate dissatisfaction [14]. Implicit in the use of the facet item is the expectation of a strong association between the overall index of job satisfaction and each of the indices of satisfaction with key facets of a job; otherwise, the validity of the instrument would be questionable.

## Objectives

Within the context of the ideas expressed in the introduction, this paper sought to explore three questions: First, do male and female pharmacists show the same levels of satisfaction both overall and with key facets of their job? Second, is the level of overall satisfaction strongly associated with the level of satisfaction with key facets of pharmacists’ jobs? And third, is the association similar for male and female pharmacists?

## Methods

This study was based on self-reported survey data. Participants were asked to assess their overall job satisfaction as well as satisfaction with key facets of their job along 0-to-10 intensity scales, with 10 denoting the greatest intensity. This procedure posed the advantage of response homogeneity as practitioners expressed their perceptions regarding various facets of their job following a common measurement standard. It provided more room for discrimination in practitioners’ responses than is normally provided by the Likert scale. Since there was no room for outliers, the mean was an adequate measure of central tendency. This scale has been used successfully to measure differentials in job-opinion variables [15, 16].

Besides overall satisfaction, 12 individual facets were included: adequacy of salary and benefits, workload, stress, advancement opportunities, job security, autonomy, fairness in the workplace, scheduling flexibility, job atmosphere, job importance to patients, supervisor’s support, and relations with coworkers. Perceived workload and stress were anticipated to detract from job satisfaction, while the other facets were anticipated to add to it. Mean indices were calculated for each of these facets and their joint variation with the overall satisfaction index was estimated using Pearson correlation coefficients. These analyses were conducted by gender and for both genders combined. The disaggregation by gender took into consideration different values previously reported in the literature for male and female pharmacists [8, 17], a disaggregation not commonly found by any other independent variable.

### Variables

Wages and salaries are the ultimate indicator of success for the American worker. A high income within the profession communicates to the world that a pharmacist is capable of coping with the pressures of the labor market and the many challenges raised by patients, employers, and peers interacting in complex ways. Thus, it is not surprising that overall job satisfaction has been linked to perceived adequacy of salary and benefits as well as intention to quit [18–20].

An excessive workload is a major source of dissatisfaction for pharmacists [21–24]; it is associated with more dispensing errors and reduced interaction with patients, which is viewed adversely by pharmacists [25]. Another source of dissatisfaction is stress [26, 27], which may lead to burnout [28, 29]; it arises when pharmacists experience unfavorable conditions over which they have no control such as role ambiguity and role conflict, unreasonable expectations from employers, and inadequate support in terms of physical or human resources [30–32].

Pharmacists’ and other professionals’ levels of job satisfaction increase with their perception of available advancement opportunities [33–35]; this is an important consideration because several studies suggest that pharmacy as a profession experiences shortcomings in satisfying the work ambitions of its practitioners [36]. Perceptions of job security also exert important effects on the pharmacist workforce through labor productivity, employment, and other outcomes and are one of the top motivating influences to enter the profession [37, 38]. Still another facet of job satisfaction is the ability to exercise one’s judgment in conducting professional activities and controlling allocation of one’s time; more discretion provides pharmacists with a sense of autonomy conducive to caring more about what they do, feeling better about it, and rendering better care to patients [20, 39].

Fairness in the workplace, also known as organizational justice, is probably the most subjective concept analyzed here for it involves not only de facto inequalities, which may be explained by market differentials, but also interpretation and feelings by pharmacists; perceived disparities in how workers are treated account for a great deal of dissatisfaction [34, 40]. Scheduling flexibility is another factor appealing to pharmacists when they compare jobs insofar as it allows them to find a better balance between the personal- and work-related aspects of their lives and reduces stressors [41–43]; part-time employment, non-standard work, flexible hours, and specialized leave policies are some of the initiatives being implemented worldwide to accommodate non-work activities [44]. Also conducive to job satisfaction (or the lack thereof) is job atmosphere, alternatively known as organizational climate [20]; it refers to employees’ perception of the typical practices and behaviors that prevail in the workplace, especially those that accord with expectations and consequently are rewarded.

The perceived importance of one’s job to patients is another contributor to pharmacists’ contentment [21, 45, 46]; interacting with patients and contributing to their wellbeing rank consistently high in the literature as intrinsic sources of satisfaction. Support from one’s supervisor also fosters contentment by virtue of increasing confidence and relieving anxiety [47–49]; according to Kahaleh and Gaither [50], this occurs when pharmacists acquire empowerment, which engenders greater organizational commitment. Good relations with coworkers is the last satisfaction-related facet analyzed here; not only do coworkers provide a dynamic and interactive background for self-expression, but they also form informal networks characterized by a horizontal flow of communication preferred by employees over channels of authority through which commands and information are transmitted downward and upward [51]. Several studies indicate that good relations with coworkers lead to job satisfaction by pharmacists [5, 26, 41].

As a general rule, average index values under 3.00 were considered low, values ranging from 3.00 to 6.99 were considered moderate, and values 7.00 and above were considered high. Similarly, Pearson correlation coefficient (absolute) values under 0.30 were viewed as indicative of a weak link, values from 0.30 to 0.69 were viewed as moderate, and values of 0.70 or higher were viewed as indicative of a strong joint variation between the overall job satisfaction index and the 12 indices hypothesized to configure job satisfaction. The values of the empirical Pearson correlation coefficients were anticipated to be above 0.70.

### Data

The survey data were gathered from responses to a questionnaire sent to 2 400 pharmacists practicing throughout the United States. These pharmacists were selected by Medical Marketing Services (MMS) using a simple random sampling scheme. MMS is a leading provider of lists of pharmacists and other healthcare professionals. Its data depository is drawn from practitioners registered with APhA, ASHP, AACP, and other organizations and includes pharmacists from all fields within the profession. Approximately 90% of the estimated 281 560 pharmacists practicing in 2012 were included in the MMS data files [52].

A survey questionnaire, previously validated and exclusively designed for this and other workforce studies, was mailed by the authors in March 2012, with a reminder sent 2 weeks later. The sample size was chosen according to Cochran [53] formula developed for categorical and other outcomes, with a 5% sampling error. The research effort was supported solely by internal university funds, and institutional review board approval was secured to conduct the probe.

### Statistical tests

Significant differences between male and female pharmacists for the overall job satisfaction index and the 12 individual indices hypothesized to configure job satisfaction were established using the *t* statistic for comparing the means of two distributions with unequal variances and unequal numbers of observations. This is a commonly used statistical test. Significant differences between genders in the Pearson correlation coefficient values for the 13 indices were established using the method developed by Fisher [54]. This procedure also was based on the *t* distribution. Since the procedure is not commonly used, its formulation is presented below:

$$ t=\frac{r{\prime}_{mi}-r{\prime}_{wi}}{\sqrt{\frac{1}{n_m-3}+\frac{1}{n_w-3}}} $$

where

$$ r{\prime}_{mi}=0.5{\mathit{\log}}_e\left(\frac{1+{r}_{mi}}{1-{r}_{mi}}\right), $$

$$ r{\prime}_{wi}=0.5{\mathit{\log}}_e\left(\frac{1+{r}_{wi}}{1-{r}_{wi}}\right), $$

*r*_*mi*_ was the male pharmacists’ Pearson correlation coefficient for the *i*th index;

*r*_*wi*_ was the female pharmacists’ Pearson correlation coefficient for the *i*th index;

*n*_*m*_ was the number of male pharmacists in the sample;

*n*_*w*_ was the number of female pharmacists in the sample; and

*i* = 1, …, 13 for the overall job satisfaction index and the 12 indices hypothesized to configure job satisfaction.

Three levels of significance (two-tail tests) were identified: *p* ≤ 0.01, *p* ≤ 0.05, and *p* ≤ 0.10.

## Results

Of the 2 400 questionnaires mailed to potential participants, 139 packets were returned undelivered for various reasons. A total of 736 pharmacists participated in the study by providing answers to all relevant questions. The numbers of observations and the response rates compared favorably with those reported by similar undertakings [4, 22, 55–57]. Of these, 436 pharmacists (59.2%) were men and 300 pharmacists (40.8%) were women. Male pharmacists’ median age was 57 years and female pharmacists’ median age was 48 years. More men (81.0%) than women (72.9%) were married and men also had, on average, more years of professional experience than women (30.6 years and 22.5 years, respectively). Approximately two thirds of pharmacists in the sample worked in the retail sector (63.0% for men and 63.4% for women).

### Indices

The means and standard deviations of the overall job satisfaction index and the facet indices hypothesized to configure job satisfaction are presented in Table 1. The values of all 13 indices were in the moderate range. Perceived job importance to patients, amount of workload, and good relations with coworkers reported the top three index values. Availability of advancement opportunities showed by far the lowest level of satisfaction, followed by perceived support from one’s supervisor and scheduling flexibility.

**Table 1:** Means and standard deviations (in parentheses) of the overall job satisfaction index and indices hypothesized to configure job satisfaction

Variables	Index values		
	Both genders	Male pharmacists	Female pharmacists
Number of observations	736	436	300
Overall job satisfaction	6.03 (3.01)	5.71* (3.22)	6.49* (2.60)
Adequacy of salaries and benefits	6.35 (2.83)	6.19^†^ (3.00)	6.56^†^ (2.57)
Workload	6.81 (2.54)	6.56* (2.68)	7.18* (2.28)
Stress	6.38 (2.91)	5.99* (3.08)	6.95* (2.52)
Advancement opportunities	3.40 (2.99)	3.21^‡^ (2.92)	3.69^‡^ (3.07)
Job security	6.00 (3.02)	5.75* (3.20)	6.36* (2.71)
Autonomy	5.88 (2.77)	5.73^†^ (2.87)	6.11^†^ (2.60)
Fairness in the workplace	6.02 (2.94)	5.92 (3.08)	6.17 (2.72)
Scheduling flexibility	5.70 (3.20)	5.40* (3.23)	6.14* (3.09)
Job atmosphere	6.17 (2.80)	6.02^†^ (2.90)	6.40^†^ (2.64)
Job importance to patients	6.95 (3.19)	6.44* (3.53)	7.68* (2.47)
Supervisor’s support	5.49 (3.28)	5.16* (3.34)	5.97* (3.13)
Relations with coworkers	6.60 (2.76)	6.34* (2.94)	6.99* (2.45)

*Statistically significant between genders (*p* ≤ 0.01)

‡Statistically significant between genders (*p* ≤ 0.05)

†Statistically significant between genders (*p* ≤ 0.10)

Male pharmacists’ overall level of satisfaction was lower than the level of female pharmacists, and the difference was statistically significant. Higher levels for women also appeared for the facet indices, and all differences were significant except fairness in the workplace.

### Joint variation

The estimated values of the Pearson correlation coefficients between the overall job satisfaction index and each of the 12 facet indices are presented in Table 2. None of them exhibited a strong joint variation and all showed a positive sign. Fairness in the workplace and job atmosphere recorded the highest values, while stress, availability of advancement opportunities, and amount of workload had the lowest values. Four of the facet indices exhibited significant differences at various levels between male and female pharmacists in their correlation with the overall satisfaction index of the same gender: the coefficients for stress and perceived job importance to patients were higher for male than female pharmacists, while the opposite was found for availability of advancement opportunities and perceived support from one’s supervisor.

**Table 2:** Pearson correlation coefficients of male and female pharmacists in the sample between the overall job satisfaction index and indices hypothesized to configure job satisfaction

Variables	Pearson correlation coefficients with the overall satisfaction index		
	Both genders	Male pharmacists	Female pharmacists
Number of observations	736	436	300
Adequacy of salaries and benefits	0.467	0.433	0.519
Workload	0.323	0.347	0.236
Stress	0.239	0.278^‡^	0.099^‡^
Advancement opportunities	0.241	0.187^†^	0.319^†^
Job security	0.504	0.486	0.524
Autonomy	0.505	0.486	0.537
Fairness in the workplace	0.615	0.598	0.652
Scheduling flexibility	0.465	0.461	0.453
Job atmosphere	0.584	0.559	0.631
Job importance to patients	0.410	0.457*	0.287*
Supervisor’s support	0.516	0.466^‡^	0.595^‡^
Relations with coworkers	0.490	0.459	0.533

*Statistically significant between genders (*p* ≤ 0.01)

‡Statistically significant between genders (*p* ≤ 0.05)

†Statistically significant between genders (*p* ≤ 0.10)

## Discussion

The empirical evidence suggests that pharmacy as a profession experiences shortcomings in satisfying the work ambitions of its practitioners, as a generalized perception of limited opportunities for career advancement seems to be prevalent. Similar findings have been reported by earlier studies in the USA and the UK [58, 59].

The finding of moderate overall and facet index values of job satisfaction also accords with the results of previous studies [36]. So does the finding of a higher overall satisfaction index value for female than male pharmacists [45, 60]. Particularly interesting was the gender differential in perceived adequacy of salaries and benefits despite the lower level of annual wages and salaries (*p* ≤ 0.01) reported by female pharmacists ($105 221) than by male pharmacists ($116 912) in the same data set. This observed incongruity is known in the literature as the paradox of the contented female worker [33, 61, 62] and is explained in terms of women having lower market expectations, feeling less social pressure at work, and internalizing their feelings of professional disillusionment to a greater extent than do men.

Also interesting was the fact that women complained more than men about the amount of workload and stress; yet female pharmacists actually worked less (37.6 h per week) than their male counterparts (40.0 h per week), and the difference was statistically significant (*p* ≤ 0.01). Availability of advancement opportunities, job security, and autonomy have been found in other studies to concern men more than women [31, 38, 63], but in this study, the opposite was the case. The relationship between the observed values of female and male pharmacists of the remaining facet indices—scheduling flexibility, job atmosphere, job importance to patients, support from one’s supervisor, and good relations with coworkers—conformed to expectations. These are considerations that traditionally affect women more than men; the greater index values obtained for female than male pharmacists reinforced the characterization of these variables as compensating differentials, that is, important work conditions for which female pharmacists showed more willingness than men to trade off earnings [7–10]. In other words, women practitioners seemed not only to place a greater emphasis on, but also be more satisfied by, social relations at work and the significance of tasks performed than men practitioners.

Contrary to expectations, both the perceived amount of workload and stress portrayed a positive joint variation with overall job satisfaction. This finding suggests that while within a specific job, an excessive workload and greater stress may detract from contentment, pharmacists generally accept that otherwise satisfying jobs require more work and stress than other jobs with fewer sources of contentment; as Gaither, Kahaleh, Doucette, Mott, Pederson, and Schommer [64] have pointed out, the practice of the profession can be both satisfying and highly stressful. In any event, the values of the coefficients were relatively low.

Perhaps the most important finding of this article was the absence of a strong joint variation between the overall job satisfaction index and the facet indices hypothesized to configure job satisfaction. Only fairness in the workplace and job atmosphere showed a correlation coefficient value higher than 0.60, both for female pharmacists, while seven indices—adequacy of salary and benefits, amount of workload, stress, availability of advancement opportunities, scheduling flexibility, job importance to patients, and relations with coworkers—showed correlation coefficients under 0.50. This seemed to question the validity of deriving a composite measure of satisfaction from various indices pertaining to key facets of a job. The empirical evidence clearly reveals that variation in the overall job satisfaction index proceeded independently of variation in nearly all individual facets. The validity of deriving a composite measure of job satisfaction was further challenged by the fact that four of the 12 facet indices exhibited significant differences between genders in their correlation coefficients with overall job satisfaction. The derivation of such a measure is likely to be flawed if differences between male and female pharmacists are not taken into account.

The findings reported here are subject to several limitations. The first limitation is that self-reported data were used, which by their very nature are open to validity and reliability criticism even though the questionnaire was tested prior to being mailed to participants. Job satisfaction is a highly subjective issue, and people’s emotions and feelings may change frequently. The study rested on a cross-sectional survey; therefore, it was inadequate to measure whether the perception of overall job satisfaction and its various facets fluctuate over time, or the extent to which such fluctuations are affected by the growth of the pharmacist workforce or the rapidly changing healthcare reform situation. Future research ought to include longitudinal data in an attempt to understand the evolving roles of the facets of job satisfaction in determining the overall picture in different time periods.

The applicability of the findings also is limited by the environmental shifts that have occurred since 2012, when the data were collected. With a focus on reducing healthcare costs and improving clinical outcomes, recent years have witnessed the development of alternative payment and delivery models such as accountable care organizations and the emergence of pharmacogenomic testing. Consequently, the role of the pharmacist continues to expand and incorporate collaborative team-based care, medication therapy management and reconciliation, preventive care services, and patient education and behavioral counseling, all of which are likely to affect perceptions of job satisfaction. Other environmental shifts include the proliferation of pharmacy schools and graduates leading to surpluses in the pharmacist workforce and the growing shift toward mail-ordered distribution of prescription drugs.

Finally, another limitation is that no incentives such as monetary compensation or raffle prizes were used to motivate survey participation; they might have altered not only the number of respondents but also the nature of the responses. Potential biases arising from the omission of facets also need be recognized. Furthermore, there was no way to corroborate that the questionnaires were received and filled by the intended respondents, which was crucial to the issue of validity.

## Conclusion

Despite its limitations, this study was successful in measuring and comparing male and female pharmacists’ overall job satisfaction as well as satisfaction with key facets of their jobs. It has identified the facets in which pharmacists reported higher and lower levels of contentment with their work, and it has shown that women were consistently more satisfied than men, even though they earned lower wages and salaries and reported greater workload and more job-related stress. It also has shown that variations in overall job satisfaction were at best accompanied by moderate variations in all facets identified in this paper as concomitants, thus raising questions about the validity of configuring a composite index from multiple indices of satisfaction with the various facets of a job. The fact that male and female practitioners differed in their observed correlation coefficient values in some facets poses additional doubts about the validity of such a procedure.

Understanding the sources and extent of job satisfaction experienced by the pharmacist workforce is likely to guide employers to prepare and alter the workplace to more adequately meet practitioners’ needs and increase their productivity. Men and women seemed to respond differently to the various facets, so a constant set of rewards and stimulants may not be equally effective for both genders. The empirical evidence reported here is expected to be used by healthcare managers and policymakers to facilitate communication, enhance teamwork, and promote a better allocation of scarce resources.

## Additional file

Additional file 1: Survey questionnaire. (DOCX 18 kb)

## Abbreviations

AACP: American Association of Colleges of Pharmacy
APhA: American Pharmacists Association
ASHP: American Society of Health-System Pharmacists
MMS: Medical Marketing Services
