# Supplementary material for: Gender differences in the measurement of pharmacists’ job satisfaction
Source: Hum Resour Health. 2018 Jul 31;16:33. doi: 10.1186/s12960-018-0297-5 (PMC6069841; doi:10.1186/s12960-018-0297-5)
Supplement: Supplementary file 1 — Survey questionnaire. (DOCX 18 kb) [file 12960_2018_297_MOESM1_ESM.docx]

**Additional file 1. Survey questionnaire**

This is a nationwide household and professional practice survey of registered pharmacists intended only for academic research purposes. You and your answers will remain anonymous at all times. Results will be reported only aggregately. Please answer all questions honestly. We thank you for your participation.

1. Gender: 2. Primary ethnic group (choose one):

[ ] Male [ ] Non-Hispanic White

[ ] Female [ ] Non-Hispanic Black (African American)

[ ] Hispanic

3. Age: __________ years old [ ] Other

4. Marital status (choose one): 5. If married, does your spouse work?

[ ] Never married [ ] Yes

[ ] Married [ ] No

[ ] Separated, divorced, or widowed

7. Place of birth (choose one):

6. Number of children you have had: ____________ [ ] Same state where you practice pharmacy

[ ] Another state within the United States

8. What academic degree(s) do you hold? [ ] Another country

[ ] B.S. in pharmacy

[ ] Pharm.D. 14. Type of primary practice site (choose one):

[ ] Other: _______________________________ [ ] Retail community/independent

[ ] Retail chain

9. Have you completed a residency or fellowship? [ ] Hospital

[ ] No [ ] Other: ________________________________

[ ] Yes. Specify: _________________________

15. Primary role as a pharmacist (choose one):

10. Do you hold any specialty board certification(s)? [ ] Clinical

[ ] No [ ] Dispensing

[ ] Yes. Area: ___________________________ [ ] Administrative

[ ] Other: ________________________________

11. How long have you been a registered pharmacist?

16. Location of your primary practice site (choose one):

__________ years [ ] Large city central

[ ] Large city suburb

12. Number of years you have practiced pharmacy [ ] Small city

[ ] Town/rural area

__________ years

1. Do you serve primarily a patient population such as

13. How long have you worked at your primary site? elderly, minority, etc.?

[ ] No

__________ years [ ] Yes. Which? __________________________

18. Distance from home to work: __________ miles 19. Average work week: __________ hours per week

20. Average one-way time commute to work: 21. Wage rate: $ __________ per hour

__________ minutes 22. Annual income from working as a pharmacist:

23. Annual household income: $ __________ per year $ ____________ per year

24. On a scale of 0-10, how satisfied are you with the profession of pharmacist?

0 1 2 3 4 5 6 7 8 9 10

Not satisfied Extremely satisfied

25. On a scale of 0-10, how satisfied are you with your current job?

0 1 2 3 4 5 6 7 8 9 10

Not satisfied Extremely satisfied

26. On a scale of 0-10, please rate the amount of work you do in relation to other workers in your place of work.

0 1 2 3 4 5 6 7 8 9 10

Least amount Greatest amount

27. On a scale of 0-10, please rate the amount of stress you experience in your job in relation to other workers.

0 1 2 3 4 5 6 7 8 9 10

Least stress Greatest stress

28. On a scale of 0-10, please rate the availability of advancement opportunities for you in your place of work.

0 1 2 3 4 5 6 7 8 9 10

Not available Most available

29. On a scale of 0-10, please rate your job security.

0 1 2 3 4 5 6 7 8 9 10

Least secure Most secure

30. On a scale of 0-10, please rate autonomy in your job.

0 1 2 3 4 5 6 7 8 9 10

Least autonomous Most autonomous

31. On a scale of 0-10, please rate overall fairness in your place of work.

0 1 2 3 4 5 6 7 8 9 10

Not fair Very fair

32. On a scale of 0-10, please rate the performance of your supervisor in helping and supporting you in your job.

0 1 2 3 4 5 6 7 8 9 10

Not at all Most helpful

33. On a scale of 0-10, please rate the job atmosphere in your place of work.

0 1 2 3 4 5 6 7 8 9 10

Least pleasant Most pleasant

34. On a scale of 0-10, please rate scheduling flexibility in your place of work.

0 1 2 3 4 5 6 7 8 9 10

Not flexible Most flexible

35. On a scale of 0-10, please rate your co-workers (*i.e.*, pharmacists, techs, etc.).

0 1 2 3 4 5 6 7 8 9 10

Lowest quality Highest quality

36. On a scale of 0-10, please rate your overall personal health.

0 1 2 3 4 5 6 7 8 9 10

Very poor Excellent
